# Understanding the influence of stakeholders on the implementation of front-of-pack nutrition labelling in the absence of public debate: the case of Portugal

**DOI:** 10.1186/s12961-023-01065-8

**Published:** 2024-02-07

**Authors:** Morgane Fialon, Lydiane Nabec, Chantal Julia

**Affiliations:** 1 Nutritional Epidemiology Research Team (EREN), Sorbonne Paris Nord University/INSERM U1153/INRAE U1125/CNAM, Epidemiology and Statistics Research Center, University of Paris (CRESS), 93017 Bobigny, France; 2 Centre de Recherche Réseaux, Innovation, Territoire et Mondialisation (RITM), Université Paris-Saclay, 91190 Gif-Sur-Yvette, France; 3 Public Health Department, Avicenne Hospital, Assistance Publique des Hôpitaux de Paris (AP-HP), 93017 Bobigny, France

**Keywords:** Front-of-pack, Portugal, Stakeholder theory, Nutri-Score

## Abstract

In the context of the European Commission’s proposal for the harmonization of front-of-pack nutrition labels (FoPLs) across the European Union scheduled for 2023, Portugal’s position on the preferred format to be adopted remains unclear. Our study aims to (1) describe the context of the political decision regarding FoPL’s implementation in Portugal, (2) identify and characterize the actors involved in the debate and (3) evaluate their influence in the decision-making process and their position on the various formats of FoPLs considered. A document review complemented by a qualitative study with eight experts revealed the actors involved in the implementation of FoPLs in Portugal. The stakeholder theory was used to qualify their level of influence on the decision. To complement the experts’ analysis, the views of 1014 Portuguese consumers on stakeholders’ influence were collected via an online questionnaire. The results show a strong mobilization of the Portuguese Ministry of Health on the issue through the production of several technical and scientific reports. The background of the political decision related to FoPLs in Portugal highlights the commitment of some influential stakeholders in favour of the traffic light format, including one of the major Portuguese retailers, Continente. Nevertheless, in the face of public support for the Nutri-Score from a growing number of actors, Portugal has not yet adopted a specific format, partly due to some resistance from the agricultural sector. Portugal, by lack of consensus and agenda setting, is now awaiting formal harmonization of FoPLs by the European Commission. This study provides useful insights for public policy actors when promoting interventional measures.

**Supplementary Information:**

The online version contains supplementary material available at 10.1186/s12961-023-01065-8.

## Background

Obesity is one of the main public health issues in Portugal among both adults (18% in 2019) and children (22% in 2018), and with higher rates than European Union (EU) average [1]. Moreover, a strong link has been identified between obesity and socioeconomic status as, in 2019, among Portuguese adults without secondary education, 22% were obese compared with 9% among those with tertiary education [1].

To tackle this health burden, Portugal has been implementing a large set of measures, especially since the creation of the National Programme for the Promotion of Healthy Eating (PNPAS) in 2012. With a focus on shifting the food environment, the following public health initiatives have been implemented: taxes on high calorific foods and soft drinks, advertising restrictions on unhealthy food products for children under 16 years old and limits on the amount of salt in bread [2]. In 2017, a joint report by WHO–Europe and the Portuguese Directorate–General for Health (DGS) showed that 60% of Portuguese with lower socioeconomic status declared they did not understand back-of-pack nutritional information [3]. The same year, PNPAS argued for the adoption of a consumer-friendly front-of-pack label (FoPL) as a strategy towards the promotion of healthier food choices by consumers, but no specific format was recommended.

At present, two main types of FoPLs coexist in Europe: interpretive formats that convey an evaluation on the nutritional value of a food (for example, the Green Keyhole in Scandinavian countries and the Nutri-Score in seven European countries) and non-interpretive formats that reproduce part of the information available on the back-of-pack without additional interpretation [4] such as the NutrInform Battery in Italy (see Additional file 1: Fig. S1).

Several of these FoPL formats can be found in Portuguese supermarkets. The reference intakes (RI) supported by the federation of the food industry in Portugal (FIPA—Federação das Indústrias Portuguesas Agro-Alimentares) is visible since 2005 on many food products [5]. The multiple traffic lights system (MTL) initially developed by the Food Standards Agency in the United Kingdom was adopted in 2009 by Continente, one of the main food retails operator in Portugal [6]. More recently, in the end of 2019, the Nutri-Score developed in France (2017) and now adopted in several EU countries has been introduced by other retail operators and food companies in Portugal (for example, Pingo Doce, Auchan Portugal, Nestlé, Pescanova) [7].

The reference intake (RI) is a non-interpretive, nutrient-specific label which displays the amounts of nutrients of concern (fats, saturated fats, sugars and salt) and energy per portion of a food product [5]. The MTL is also a nutrient-specific FoPL but with an additional evaluative information where each color is associated with the nutrient amount: red for a high amount, amber for a moderate amount and green for a low amount [6]. Finally, the Nutri-Score is both an interpretive and summary indicator with five categories from dark green/A to dark orange/E attributed on the basis of a nutrient profile model considering, for 100 g or 100 mL of product, the content of nutrients to be limited and of nutrients and foods to be favoured [7].

In the context of the harmonization of FoPLs by the European Commission foreseen in the “Farm to Fork strategy” [8], Portugal government has not expressed any public position on the format to be implemented. In parallel, some local actors have manifested their support to Nutri-Score such as the main consumer association Deco Proteste [9], some scientists, some distributors (for example, Auchan Portugal, Pingo Doce) and some food companies (for example, Nestlé Portugal, Danone Portugal, Pescanova).

In the public health sector, economic operators are known to influence governmental decisions. Theories such as the commercial determinants of health (CDoH) or the corporate political activities (CPA) have shown the various ways in which the unhealthy commodity industries (UCIs) (for example, tobacco, ultra-processed foods and beverages) can impact policy-making by accessing and influencing policy-making, using the law, manufacturing support for industry, shaping evidence to manufacture doubt, displacing and usurping public health, and managing reputations to the advantage of industry [10, 11].

However, less research has explored the role of other stakeholders in the implementation of a public health measure such as FoPL, especially considering its multisectoral facet. Compared with other countries, such as Italy [12], there has been no strong opposition from the Portuguese food industry to interpretive FoPLs; on the contrary, several companies have publicly expressed their support for Nutri-Score. This context has led us to adopt a broader analytical framework. Indeed, although Portugal has included the implementation of an interpretative nutritional label in its “Integrated Strategy for the Promotion of Healthy Eating” (EIPAS, 2017), several labelling systems still coexist on the Portuguese market. This study aims to identify the underlying reasons leading to this situation focusing on three main objectives: (1) to describe the context of the political decision on the issue of FoPL in Portugal and its construction, (2) to identify and characterize the stakeholders involved in FoPL implementation in Portugal and (3) to evaluate the stakeholders’ influence in the decision-making process. To achieve these research goals, we confront Portuguese public health experts with consumers perceptions on the level of influence of the stakeholders involved in FoPL implementation in Portugal.

## Theoretical background

Sociology and political science refer to a problem as a situation perceived as problematic by the people more or less directly concerned or by groups that are involved in denouncing it [13]. The Portuguese government officially recognized the problem of overweight/obesity and diet-related chronic diseases in Portugal by, notably, creating in 2012 the PNPAS to educate and inform the population on nutrition and by introducing regulations aiming at curbing the obesity epidemic, including the need of an interpretive FoPL in Portuguese nutritional guidelines. However, this observation did not lead to the adoption of an official FoPL at a national level. When the importance of a public problem is recognized by institutions and solutions are identified, state intervention implies that the political decision be put on the agenda to be taken (draft law, parliamentary discussions and vote) and then implemented by state services. The prioritization of policy decisions is thus a key step that emanates not only from state policy but also from the mobilization of actors in the field and the media. The prioritization allows the political decision to be made based on the available knowledge and expertise of the actors. When the political decision is not put on the agenda, the situation is not a priority, and it may be referred to as a nonproblem [13]. In a nonproblem situation, issues are depoliticized and the debate is not carried into the public arena, the power relations remaining invisible. In Portugal, the decision to implement an FoPL was not on the agenda during our study and the debate was not carried into the public arena except by very few stakeholders, unlike in other neighbouring European countries such as Italy or Spain [12, 14]. To understand the influence of stakeholders on the implementation of a FoPL in a context of nonproblem, it is necessary to characterize its historical construction, leading to a political nondecision.

Regarding the role of stakeholders external to the government, E. Henry states that media can act as resonance chambers to bring problems to wider spaces and to reach larger audiences [13]. In addition, according to their power and their representation in certain ministerial sectors or in the parliament, some actors can directly influence the spheres of public decision-making without being visible in the public space when less powerful ones must use the public space to impose their cause and put pressure on the government. This calls for a precise identification and characterization of all the stakeholders involved in the implementation of a FoPL in Portugal.

**Fig. 1:**
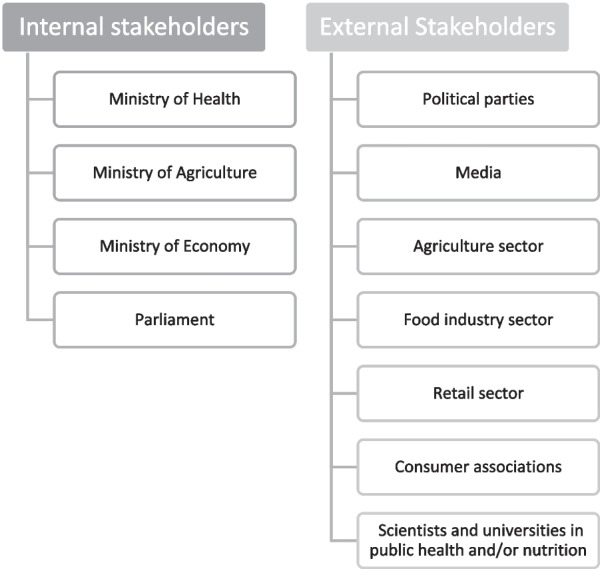
Main categories of internal and external stakeholders involved in FoPLs implementation in Portugal

Freeman (1984) explained the concept of stakeholder with the following definition: “a stakeholder in the organization is [by definition] any group of individuals or any individual who can affect or be affected by the achievement of organizational goals” [15]. This definition is part of the “stakeholder theory” (SHT), which emphasizes that a company must consider and create value for all the “stakeholders” in its organization, that is, its customers, suppliers, employees, investors and so on, to ensure its smooth operation. In addition, based on the work by Caroll and Näsi (1997), stakeholders can be classified into “internal”, that is, within the company (owners, managers, employees) and “external”, such as competitors, consumers, governments, pressure groups, the media, the community or the natural environment [16]. We transposed this classification intended for a private company to our case study with internal stakeholders being the Ministry of Health, Ministry of Agriculture, Ministry of Economy and Parliament, and external stakeholders being political parties, media, the agriculture sector, the food industry sector, the retail sector, consumer associations, scientists and universities (Fig. 1).

Several variations on SHT have emerged over the years [17–19, 23]. As developed by Mitchell, Agle and Wood in 1997 [20], the SHT has been mobilized in social marketing research to understand how consumer associations could strengthen their influence in protecting consumer interests [18]. It thus provides a relevant framework for studying the stakeholders involved in the implementation of a FoPL in a country [12]. Indeed, Mitchell et al. added the salience framework, which allows the classification of stakeholders on an ordinal scale, relatively to one another by following three steps.

The first step is to identify the stakeholders who can affect or be affected by the achievement of the goals, that is the decision of implementation of a FoPL in the country. Then, the second step is to qualify the attributes of power, legitimacy and urgency of them leading to its classification in one of the eight groups defined by the authors [20]. Power is defined as: “the ability of a stakeholder to act to obtain the decisions they want” [18, 20]; legitimacy is considered as: “the general perception that the actions of the stakeholder are desirable, adequate or appropriate within a system of beliefs, values and social norms”. [18, 20, 21] Finally, urgency captures “the critical nature of the stakeholder’s claims and the immediacy with which the firm is required to respond to them”. [18, 20]. As a third step, the salience of a stakeholder can be defined from high to low according to the number of attributes each stakeholder possesses. The most salient stakeholders are those who have the power to act, the legitimacy to do so and the capacity to act in an emergency. In other words, the salience corresponds to the degree to which decision-makers place priority on competing stakeholder claims [22]. In this study, instead of “salience” we used the term “influence” which has been also used in in the study by Varvasovsky et al. [23] as we found it more intelligible for the reader.

Recent research shows that the SHT theoretical framework developed by Mitchell, Aggle and Wood [20] has demonstrated its growing acceptance and utility as a tool for identifying and prioritizing stakeholders in various fields [22]. However, Khurram et al. [22] point out three key themes to improve the understanding of stakeholder’s salience. In particular, it is necessary to study the status of stakeholders to better prioritize their claims and to take into account the contextual factors that influence the pragmatic power of stakeholders in the situation, and its dynamic. Actually, a stakeholder can hold power at one point in time but might not possess the same power at another time, or a stakeholder might possess more power at one point in time but less at another. Therefore, the contextual analysis may also consider the interdependent relationships among stakeholders in coalitions.

In our case, public health measures such as the adoption of a FoPL are implemented over a relatively long period of time and, since this measure is rather related to prevention, stakeholders demand rarely require immediate attention from the government compared with other health topics. Additionally, several authors show that the attribute of urgency can be ambiguous [22]. For these reasons, we decided not to include the urgency in our analysis. Additionally, controversies over the solutions to be implemented result in opposing positions taken by the various stakeholders, which it is necessary to characterize to understand their influence on the decision. In consequence, to evaluate more precisely the influence of each Portuguese actor in the implementation of a FoPL, we considered their position and visibility on the issue [23]. Position assessed whether the actor was supportive, neutral or in opposition to the implementation of Nutri-Score in Portugal. A position towards the Nutri-Score specifically was selected as it appeared to correspond to the latest development in the FoPL debate in Portugal. The visibility was measured though the number of experts who thought the actor was part of the debate or not, reflecting as well the level of consensus on the inclusion of stakeholder in the debate among the experts interviewed.

This theoretical framework enables us to apply the stakeholder theory more appropriately to a public health issue such as a country’s implementation of a FoPL. Moreover, to address limitations identified by Khurram et al. [22], we propose three main additional perspectives:

- the consideration of the historical perspective of the political decision to elucidate the positions of the various stakeholders at the time of the study, their dynamics and the alliances that may have existed
- the classification between internal and external stakeholders to better capture the multisectoral aspect of the topic and to refine the study of influences and positions within a government
- the comparison of expert opinion on the influence of stakeholders with that of consumers to better identify the potential discrepancy between their perceptions and the influence it may have on the implementation of a FoPL.

## Material and methods

### Document review

To analyze the context of FoPLs implementation in Portugal as well as the construction of the nonproblem situation, we conducted a document review via three main sources: scientific papers; grey literature (technical reports, legislation documents and so on) and generic online press. The document review focused on the last decade 2009–2021 which reflected the period where FoPLs started to appear in the Portuguese supermarkets with the adoption of the MTL system by the retail brand Continente in 2009 and when the Portuguese government officially recognized FoPLs as a solution to tackle overweight and nutrition-related diseases and produced technical reports on the issue. To identify scientific papers on the topic of FoPLs involving Portuguese authors, we used the platform Google Scholar. The use of the keywords “front-of-pack nutrition label Portugal” with a date selection from 2009 to 2021 gave a total of 728 results. The use of the keywords “Nutri-Score Portugal” with a date selection from 2009 to 2021 gave a total of 208 results. After selecting scientific papers which included at least Nutri-Score and were cosigned by at least one Portuguese author, three main scientific papers emerged [24–26].

Grey literature on the topic of FoPLs in Portugal were mainly indicated by the experts in the interviews conducted. Also, to identify the main political decisions related to FoPLs and nutrition in Portugal, two main scientific papers were used: A New Interministerial Strategy for the Promotion of Healthy Eating in Portugal: Implementation and Initial Results [27] and A Decade of Food and Nutrition Policy in Portugal (2010–2020) [28].

For the press articles, we searched for the keywords “Nutri-Score Portugal rotulagem nutricional” by selecting a period from 2009 to 2021 on Google News. A total of 33 articles resulted from this online request.

**Fig. 2:**
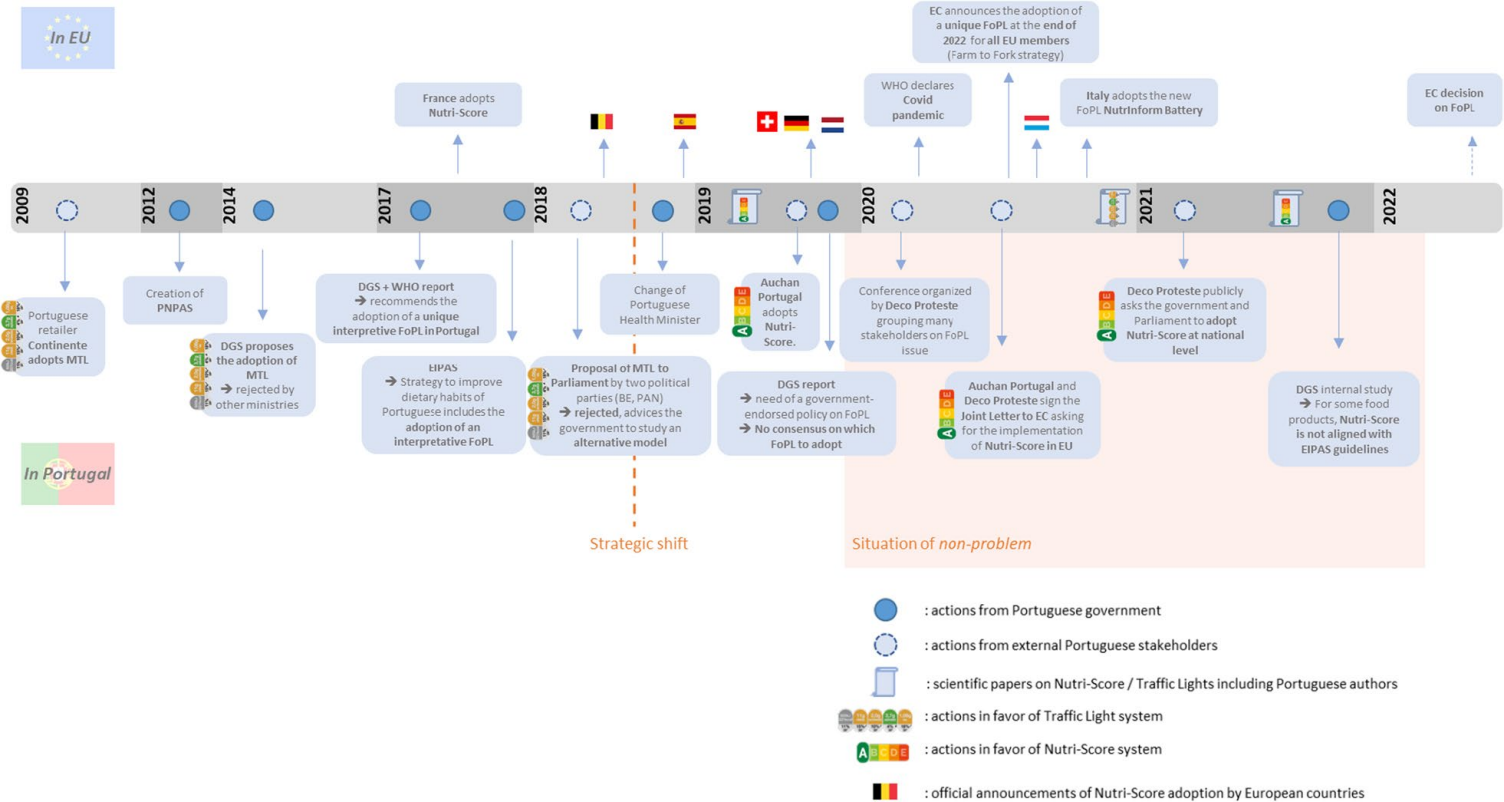
Main strategic documents and historical markers in the field of front-of-pack labelling for the period 2009–2021 in Portugal and Europe. *WHO* World Health Organization, *DGS* Directorate-General of Health, *PNPAS* National Programme for the Promotion of Healthy Eating in Portugal, *EIPAS* Integrated Strategy for the Promotion of Healthy Eating in Portugal, *BE* Bloco de Esquerda (left-wing socialist political party), *PAN* Pessoas-Animais-Natureza (environmentalist and animal rights focused political party), *FoPL* Front-of-Pack Label, *MTL* Multiple Traffic Lights system

All documents were archived using Zotero and classified according to their nature and date of publication, before being summarized (Additional file 1: Table S1). Indeed, the key dates and key facts relating to FoPLs in Portugal were then used to create Fig. 2. Some documents also enabled us to refine the position of certain stakeholders.

### Experts interviews

To complement the document review, we contacted several Portuguese nutrition and public health experts, soliciting an interview on the topic of front-of-pack labels in Portugal, and more specifically on the actors directly involved in the implementation of FoPLs in Portugal. In the end, we conducted semi-directive interviews with eight experts from May 2021 to July 2022. Experts were related to a wide range of structures in both private and public sectors involved in FoPLs debate (Table 1).

**Table 1:** Expert characteristics

Name in text	Nationality	Workplace type	Experience	Interview date	Interview length
Expert 1	Portuguese	Portuguese consumer association	Senior	26/05/2021	1 h 13 min
Expert 2	Portuguese	Portuguese major food company	Senior	27/05/2021	1 h 08 min
Expert 3	Portuguese	Multinational food company	Senior	28//07/2021	1 h 20 min
Expert 4	Portuguese	Multinational food company	Senior	28/07/2021	1 h 20 min
Expert 5	Portuguese	Portuguese major food retailer	Senior	01/09/2021	0 h 58 min
Expert 6	French	French nutrition research structure	Senior	30/03/2022	0 h 54 min
Expert 7	Portuguese	Nutritionist, EU Parliament	Junior	21/01/2022	1 h 22 min
Expert 8	Portuguese	Portuguese Ministry of Health (DGS)	Senior	19/07/2022	0 h 55 min

The interviews were structured in the following way: (1) general public health issues in Portugal, context; (2) characterization of stakeholders’ power and legitimacy; and (3) opinion of the expert on FoPL formats (Additional file 1: Table S2). All the interviews were conducted online, recorded and then fully transcribed.

The first expert was presented with a preliminary list of Portuguese stakeholders retrieved via the document review. For the following interviews, respondents were asked to identify all other important stakeholders who had, or could have, influence in the implementation of a FoPL in Portugal to complement the list [29]. After defining the notions of power and legitimacy to the expert, they were asked to grade these attributes between “1” and”3”, the number “3” being the highest level of the attribute (for example, high power) and “1” the lowest level of the attribute (for example, low legitimacy). Indeed, taking into account the previous work done by Roux et al. [18], we decided that the attributes would be evaluated by the experts on a discrete scale (from 1 to 3) instead of a dichotomous evaluation (yes/no) to allow a finer reading. Experts were free to skip some stakeholders if they thought there were not relevant in the context of the FoPL debate in Portugal. Following the interview, the grid was redrafted and then sent back to the expert for validation. The final grids of the eight experts were then aggregated to obtain an average power and legitimacy mark for each stakeholder. Visibility of a stakeholder, defined as the number of experts who mentioned the stakeholder in the interview, was also included. We then added the position of the stakeholder on Nutri-Score (in favour, neutral or against) according to the experts verbatims and the document review.

To obtain a more synthetic vision, we grouped stakeholders into categories such as political parties or food industry sector. Power, legitimacy and visibility per category corresponded to the average of these attributes among all the stakeholders inside the category. Position of a category was assessed according to the majority of stakeholders’ positions, the option “mixed positions” was added in the case of conflicting views on the topic. All these attributes allowed us to characterize the influence of a stakeholder in the implementation of a FoPL in Portugal, which was driven mainly by the power attribute [23].

In parallel of the grid analysis, we performed a content analysis based on the interview transcriptions by classifying the verbatims according to the designated stakeholder. The most relevant verbatims were then selected and included in the result part.

### Evaluation of stakeholders by Portuguese consumers

To complement the analysis of experts, we retrieved the point of view of Portuguese consumers on stakeholders’ power and legitimacy in the frame of the implementation of a FoPL in Portugal. Between 6 May 2022 and 28 June 2022, 1014 Portuguese participants were recruited by the International Organization for Standardization (ISO)-accredited international web panel provider PureProfile, to perform an online questionnaire on the topic of FoPLs. This specific sample allowed us to approach quotas on age, sex and education level of the general Portuguese population (mean age = 45.1 ± 13.8 years old, 49% women, 39% with a university degree, 32% with a children ≤ 13 years old) [30]. Portuguese participants had to give their opinion on eight categories of stakeholders selected according to the document review and the first experts’ interviews: scientists and researchers in public health and/or nutrition, consumer associations, Ministry of Health and public health institutions, Ministry of Agriculture and other institutions related to agriculture, media, retail sector, food industry sector and political parties. For each category of stakeholder, the consumer had to give their opinion on six statements assessing their power and legitimacy through a seven-point Likert scale from “1 strongly disagree” to “7 strongly agree” with “4 neither agree nor disagree”. The statements derived from Mitchell et al. for the power attribute [20] and Litchtlé et al. [31] regarding the legitimacy are presented in Additional file 1: Table S3. Of note, since the power and legitimacy rating scales differed between experts (1–3) and consumers (1–7), we only compared stakeholder rankings on these attributes.

## Results

### Towards a context of nonproblem in Portugal

This timeline (Fig. 2) can be divided in two main periods regarding the implementation of FoPLs in Portugal, with a turning point in 2018. The first period corresponds to a global dynamic towards the adoption of the MTL system with several proposals from the government or political parties brought forward to the parliament but with no adoption obtained. The second period starts after parliament’s rejection of MTL implementation in Portugal with the recommendation to study the potential of other FoPLs in April 2018. While the Nutri-Score is being adopted in several EU countries, DGS evaluates different FoPLs’ potentials but no system emerges as the preferred one. After the publication of this report in December 2019, the situation on FoPLs in Portugal moves slowly towards a situation of nonproblem, despite the pressure of external stakeholders, with no agenda setting of the measure by Portuguese government leading to a nondecision.

#### First period: 2009–2018

Since 2005, several front-of-pack labels have been gradually introduced in Portugal by economic operators. Although the reference intakes label was the first visible on some food products in Portugal, Continente was the first Portuguese retailer to officially adopt a FoPL. Indeed, in 2009, the major Portuguese retailer Continente part of Sonae group (25% of market share, 2013 [32], Additional file 1: Table S4) decided to use an adapted version of the multiple traffic light system (MTL; UK Food Standards Agency) on all of its Continente-branded food products independently from governmental guidelines.

In parallel, national nutritional programmes were defined in Portugal. The PNPAS – Programa Nacional de Promoção da Alimentação Saudável [2] was created in 2012 under the supervision of professor Pedro Graça inside the Ministry of Health (Direção Geral da Saúde – DGS). In 2014, DGS discussed the adoption of the MTL system at the national level successively in 2014 and then in 2016, without finding approval [27]. One of the arguments put forward by the opposition was that back-of-pack nutritional information was sufficient to guide consumers’ food choices. To test the validity of this statement, DGS with the support of WHO Europe, conducted in 2017 a study entitled Portuguese consumers’ attitudes towards food labelling, showing that 40% of Portuguese participants did not understand back-of-pack nutritional information. This figure was even higher (60%) for participants with lower socioeconomic status [3]. Moreover, this study showed that MTL was the preferred scheme for all focus groups. However, the report did not conclude on a specific format, as the recommendation was to adopt an interpretive FoPL that was easy to use for consumers at the national level.

In December 2017, a new interministerial strategy was published as a law under the name of EIPAS (Integrated Strategy for the Promotion of Healthy Eating). EIPAS included four strategic intervention areas among them the development of an interpretative front-of-pack nutrition label (Strategic area 2 – improve quality of and consumer accessibility to healthy food choices).

In parallel, MTL adoption proposals were brought to parliament successively by two political parties (BE and PAN) [33, 34] in 2017–2018 but were then rejected. The Portuguese parliament recommended the assessment of alternative FoPLs to the government in April 2018 [35]. This recommendation represented a turning point in the political decision process on the implementation of a FoPL in Portugal. Indeed, with the arrival of Nutri-Score in the European landscape, the government began to consider the Nutri-Score format.

#### Second period: 2019–2021

In March 2019, a scientific paper cosigned by Portuguese and French scientists, concluded that “Nutri-Score would be an adequate FoP labelling system to be considered and endorsed by Portugal” [25]. In December 2019, various FoPL options were studied by DGS in the report entitled Improving Nutrition Labelling In Portugal – Health Impact Assessment [36] which did not conclude on a specific superior FoPL. With regards to the Nutri-Score, the report highlighted some concerns with the algorithm and the need for future studies focusing on its improvement and its suitability with Portuguese food products and national nutrition guidelines.

While the Nutri-Score was in the process of being adopted in several other EU countries, no decision came from Portugal in the following years, leading to a situation of a nonproblem. However, a few external Portuguese stakeholders kept bringing the topic into the public space. In February 2020, the consumer association Deco Proteste organized a conference on FoPLs [37], in which pro-MTL stakeholders, such as the Sonae group, confronted those in favour of Nutri-Score, again without reaching a consensus [38]. DGS stated that they would support any simplified nutrition labelling system [38], thus postponing a formal decision on the issue. In May 2020, some European stakeholders sent a joint letter to the European Commission asking for Nutri-Score to be mandatory in EU. Among them, we could find two Portuguese actors: the consumer association Deco Proteste and Auchan Retail Portugal. At the same time, the implementation of a harmonized FoPL became a European issue as it was included in the EC Farm to Fork Strategy [8]. A few months later, a different team of Portuguese scientists (compared with the team of the previous scientific paper in favour of Nutri-Score mentioned before), published a paper finding that MTL was preferred and performed better on objective understanding compared with Nutri-Score [26].

In parallel, the Nutri-Score began to appear in Portuguese supermarkets through international companies (Nestlé, Danone and so on) but also national brands (for example, PescaNova) and among retailers (Auchan, Aldi, Pingo Doce and so on). In May 2021, Deco Proteste launched a campaign in support for Nutri-Score called “Nutri-Score no Rótulo”, publicly asking the Portuguese government to adopt it as a national scheme with no reaction obtained from the government. At the same time, DGS released an evidence report in which the Healthy Food Environment Policy Index (Food–EPI) was used to assess the level of implementation of public policies in Portugal [39, 40]. With regard to the existence of a FoPL policy (part of the Food–EPI indicators), the report gave an overview of the Portuguese and EU situations without recommending any particular format.

Finally, DGS conducted an internal study published in December 2021 where Nutri-Score was applied on a sample of Portuguese food products. They concluded that a majority of products well classified by Nutri-Score exceeded the nutritional values in salt and sugar as defined in the national Portuguese guidelines (EIPAS – Integrated Strategy for Promoting Healthy Eating) [41]. Again, conclusions of this study did not lead to a specific FoPL recommendation for Portugal. Of note, in 2022, the Minister of Health changed in Portugal.

While the analysis of the history of FoPL facts in Portugal has explained in part why this topic has not become a public problem, it is necessary to understand the influence of the external actors, especially the ones who do not need the public space to reach the government. This led us to the next part of the results obtained from interviews with experts directly involved in the implementation of FoPLs in Portugal.

### The stakeholders involved in front-of-pack implementation in Portugal and their influence

**Fig. 3:**
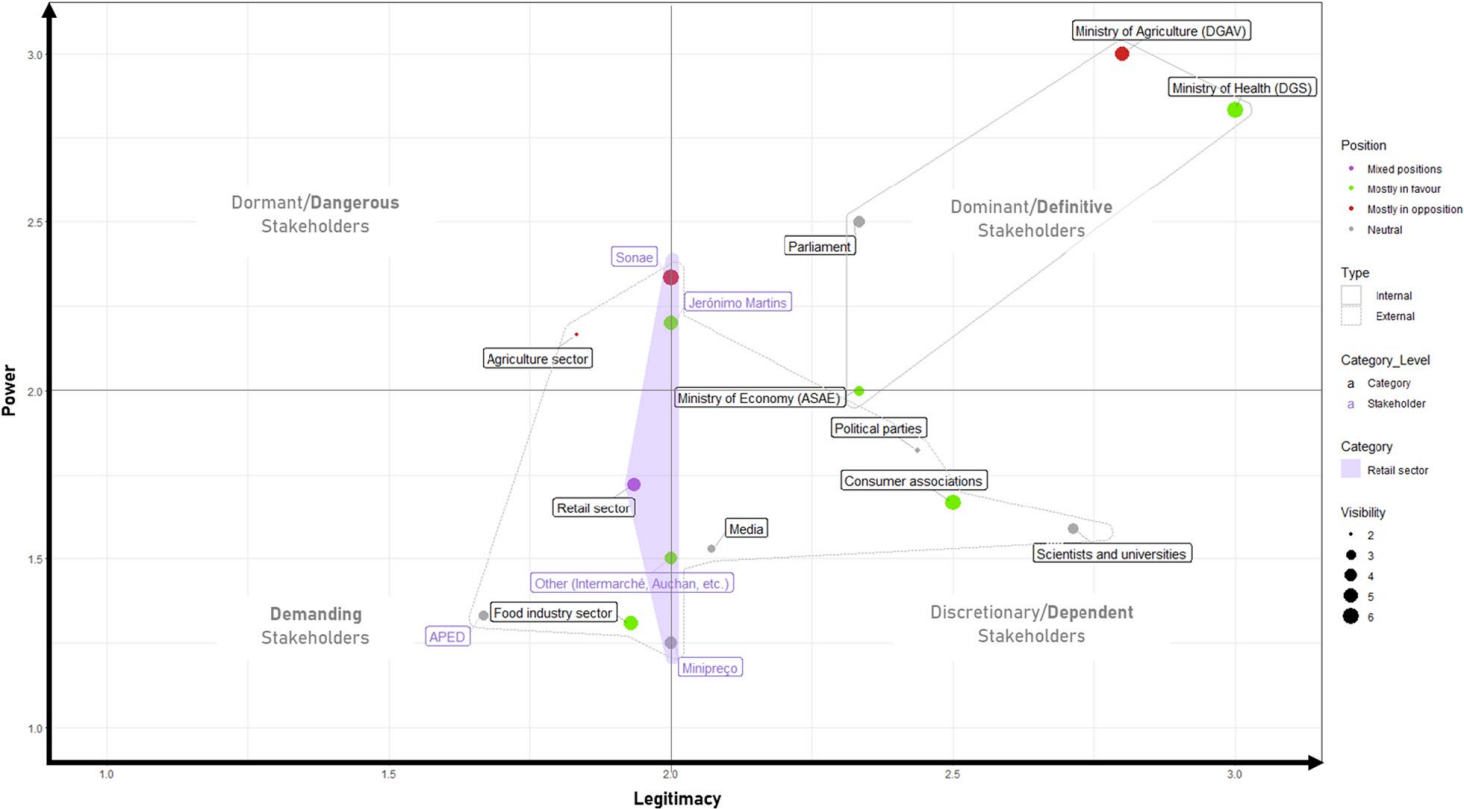
Stakeholders’ power, legitimacy, visibility and position on Nutri-Score’s debate in Portugal and classification according to the stakeholder theory

From the 42 stakeholders initially identified during the document review, the interviews conducted with the eight experts selected for the study brought the total number of stakeholders to 68. External stakeholders were classified in seven categories, while for the internal stakeholders, we displayed only the ones identified as playing a major role in the implementation of a FoPL in Portugal (Fig. 3).

#### Internal stakeholders: a strong mobilization of the Ministry of Health on the issue and a steady opposition of the Ministry of Agriculture to FoPLs

Nutrition policies in Portugal are managed by the Ministry of Health, except for food labelling issues which fall under the scope of the Ministry of Agriculture (DGAV) and food safety policies which falls under the mandate of the Ministry of Agriculture (DGAV) and the Ministry of Economy (ASAE) [42]. The national food safety authority (ASAE) is responsible for the enforcement of food safety laws set by DGAV. DGS, which is a public body of the Ministry of Health, is responsible for the National Programme for the Promotion of Healthy Eating (PNPAS) and supervises health-promotion activities in Portugal. DGS also provides technical support for the Health Secretary of State. DGADR, another public body inside the Ministry of Agriculture, is focused on agriculture policies but it is also responsible for the promotion of the Mediterranean diet and traditional products. Finally, the Portuguese parliament is formed of a single chamber of members and is called the *Assembleia da República*.

**Fig. 4:**
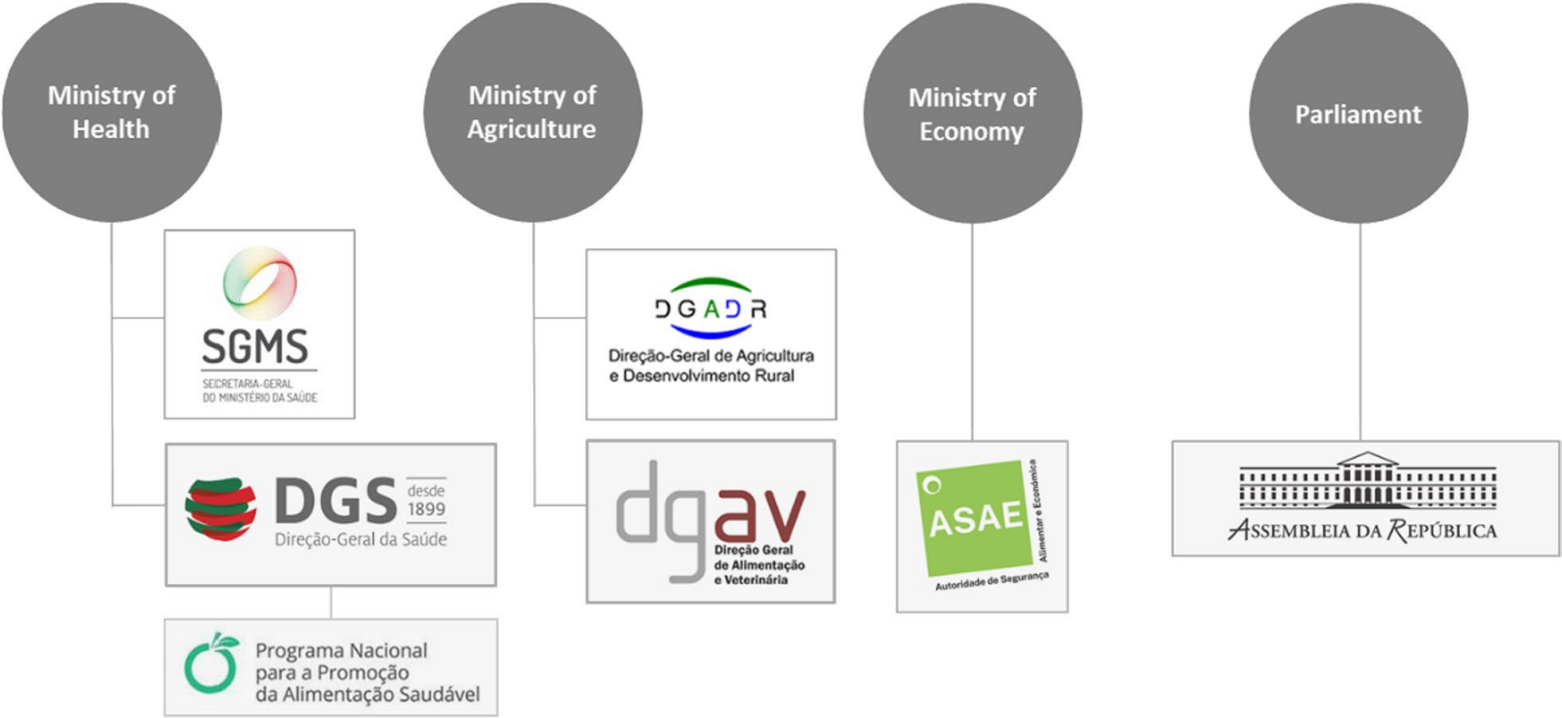
Main internal stakeholders related to FoPLs implementation in Portugal

According to the experts, the four main internal stakeholders involved with FoPL implementation in Portugal (Fig. 4) were the following (starting with the stakeholder with more power):

- *The Direção-Geral da Alimentação e Veterinária* (DGAV), a part of the Ministry of Agriculture
- *The Direção Geral de Saúde* (DGS), a part of the Ministry of Health
- *The Assembleia da República*, as the Portuguese Parliament,
- *The Autoridade de Segurança Alimentar e Económica* (ASAE), a part of the Ministry of Economy

Their power to influence front-of-pack’s implementation varies. DGAV and DGS were the stakeholders with the highest power, legitimacy and visibility in the debate on FoPLs in Portugal based on experts’ interviews:

"DGS and DGAV, among the political bodies, are the main actors who decide whether or not to implement the Nutri-Score." Expert 1

As DGAV is the public entity responsible for the implementation of policies related to food labelling, experts attributed it more power compared to DGS:

"DGAV has power, legitimacy, supervises everything, regulates everything (reg 1169), has the final decision, advises the prime minister. DGS doesn’t have the full power but provides the advices to DGAV." Expert 3 & Expert 4

"DGAV are the keepers of food law and food labelling, they make the link with European institutions, they are closer to the food industry, the DGS can’t impose a law. They tried but it’s not possible to impose a FoPL without DGAV." Expert 2

The next highest stakeholder in terms of power was the Portuguese parliament, by which a measure such as the implementation of a front-of-pack label must be approved. Finally, expert 8 mentioned that interministerial groups working on the topic of FoPLs included also ASEA, the national food safety authority part of the Ministry of Economy.

In terms of legitimacy, DGS responsible for the National Programme for the Promotion of Healthy Eating (PNPAS), obtained the highest average legitimacy score among experts:

“PNPAS stands still as the most legitimate source” Expert 7

DGS was mentioned as being concerned by the topic of FoPLs by expert 1:

"DGS has been worried about this question of FoPL, in fact they are quite active now related to that"

In terms of positions on Nutri-Score, since no public declarations from the government were identified in the media, interview with expert 8 from the government allowed us to define the status of each stakeholder:

"DGAV (Ministry of Agriculture) usually aligns with the positions from the agriculture sector. There is the processed meat sector, we have production in this area in Portugal and the olive oil one that is probably another sector that is not favourable to Nutri-Score, but mainly processed meat, I think, and traditional products are a problem.

The Ministry of Economy, I think today supports Nutri-Score because we have big companies in the corporate sector and retailer sector that support this FoPL, so now they are more favourable than in the past. [...] So I think the debate is not as visible because we have different positions between ministries, so it is more difficult to provide a public position in this area.

DGS (Ministry of Health) supports the implementation of a FoPL. In fact, we tried to implement the MTL in the past and it was not approved by the parliament. We tried with different approaches, but each time it was rejected. In fact, we had also proposed to implement Nutri-Score, but again we did not get approval from all the ministries. So, for the MTL, only the Ministry of Health supported it, while for Nutri-Score, we have the Ministry of Health and Economy, but still not the Ministry of Agriculture."

According to the experts, DGS, DGAV, the Parliament and ASAE represent dominant/definitive stakeholders [20] in the scope of the implementation of a front-of-pack nutrition label in Portugal as they have the power and legitimacy to decide the format of FoPL to be implemented in Portugal.

#### External stakeholders

##### Dormant/dangerous stakeholders: high power, low legitimacy

The first type of actors that emerged from experts’ evaluations of power and legitimacy attributes are dormant/dangerous stakeholders which have a high power but a low legitimacy. At the level of actor categories, the agricultural sector was the only category in the dormant/dangerous stakeholders’ group.

Despite its low visibility in the Portuguese debate on FoPLs, the agriculture sector represented by the Confederation of Farmers in Portugal (CAP) and some traditional food consortium such as *Casa do Azeite* (olive oil sector) seemed to influence the Ministry of Agriculture in its opposition to Nutri-Score:

“The Federation of Portuguese Agri-Food Industries (FIPA) and the Portuguese Association of Retailing Companies (APED) are more involved in this discussion because they have more processed foods than the Confederation of Farmers in Portugal (CAP). However, CAP doesn’t really support any FoPLs. The Ministry of Agriculture represents CAP. So, CAP is not so involved in the debate but they still have an influence”. Expert 8.

“The traditional product sector has power like the wine one, CAP has power even more than FIPA but I don’t know if they will express themselves on FoPLs, until now we didn’t hear them”. Expert 2.

“In June I will participate in a congress where there will debate about FoPL, the people invited are more opposed to Nutri-Score (Sonae, Industry of olive oil and cheese), I don’t think it will go much in the sense of supporting Nutri-Score. […] in Portugal we haven’t heard from the olive oil industry yet like in Spain”. Expert 1.

Although the Portuguese experts considered that the agriculture sector had some power to influence the implementation of a FoPL in Portugal, it is interesting to note that they attributed the lowest average legitimacy to this category of stakeholder. The agriculture sector did not communicate widely its position in the media, which could also explain its low visibility resulting from the experts’ interviews.

However, a public statement by the general secretary of Casa do Azeite – Mariana Matos – was published in an online specialized magazine [43]. As a stakeholder that would be impacted by FoPLs implementation, the traditional food consortium recognized the need of a harmonized FoPL in EU but did not support the current algorithm of Nutri-Score in the particular case of olive oil:

“Caza do Azeite has welcomed the European Commission’s intention to regulate the FoP nutrition labelling, to avoid the proliferation of schemes that only fragment the market and confuse consumers. But it considers indispensable a revision of the algorithms that allow ‘recognizing’ the nutritional value of a product like olive oil, which is a 100% natural mono-ingredient product with dozens of compounds of high nutritional value, which count zero in the calculation of the ‘nutritional value’ in the proposed schemes, namely the Nutri-Score. If this is not possible, then it should be considered that some natural products, which do not allow reformulation, may be exempted from the obligation to declare them, although we believe that this may be an unfavourable situation for the consumption of olive oil”. [43]

##### Discretionary/dependent stakeholders: low power, high legitimacy

The second type of external stakeholders had a lower power to act on the implementation of a FoPL in Portugal or to influence it compared with the internal stakeholders and the agriculture sector but had a high legitimacy on the issue. They are considered as discretionary/dependent stakeholders. We have identified four categories of discretionary/dependent stakeholders: political parties, consumer associations, scientists and the media.

The first category includes political parties which were considered by the experts as having the highest power to act to implement a FoPL in Portugal. Indeed, the previous part on historical facts showed us that political parties could propose FoPLs implementation directly to Parliament. In 2018, the Left Bloc (BE) and the People Animals Nature (PAN) were the two parties that proposed the adoption of MTL to the parliament [33, 34]. To the best of our knowledge, no other Portuguese political party had taken specific action to implement FoPL during this period. The political parties identified as having a specific interest in FoPL by the experts were PAN, BE and Livre:

“PAN (People Animals Nature), BE (Left Bloc), Livre (eco-socialist political party) would probably be interested in FoPL topic”. Expert 8.

“I would say PAN as being the more, the most likely to talk about Nutri-Score because you know, PAN focuses a lot on the common agricultural policy, Farm to Fork. So, they talk a lot more about nutrition related aspects compared to most parties like vegetarianism, what is a sustainable eating, etc. So, they would go around it”. Expert 7.

However, regarding Nutri-Score no public declarations or specific positions from political parties were identified by the experts explaining their low visibility on the topic. The main consumer association in Portugal, Deco Proteste, was the second discretionary/dependent stakeholder in terms of power ranking. However, it was mainly characterized by its high visibility in the debate as it was mentioned by all the experts interviewed. The mobilization of Deco Proteste on the issue was highlighted by the experts:

“Deco Proteste has a strong influence on the government, they can put a sense of urgency on the topic, but they are not the one to take the decision”. Expert 3 and expert 4.

“They have done a lot of campaigning. They organized a meeting quite a few years ago to try to convince a number of actors. Deco Proteste has done awareness campaigns with parliamentarians, there have been a lot of actions developed on the consumer side, there is a great consistency in their positioning”. Expert 6.

Deco Proteste became publicly in favour of Nutri-Score by signing the joint letter addressed to the European Commission in May 2020 [44] and organized two main campaigns asking the Portuguese government and parliament to adopt Nutri-Score:

“We did one campaign in 2019 where we started introducing Nutri-Score to the population, at this time they were very few products with Nutri-Score. Then, we launched on the 1st of May 2021 the other campaign called ‘Nutri-Score no Rótulo’ to ask for the implementation of Nutri-Score in Portugal, we sent letters to DGS, DGAV, the Health Secretary of State and the Professional Order of Nutritionists. We received a lot of support from consumers and we have partnerships going on with Auchan, Danone and Nestlé”. Expert 1.

The experts attributed quite a high legitimacy to the consumer association; however, their use of the public space to impose their cause tends to be characteristic of actors that do not have sufficient resources to directly influence the spheres of public decision-making which explain their relatively low overall power on the issue.

The power of scientists to act for or to influence the implementation of a FoPL in Portugal was mainly related to the degree of influence of the stakeholder of which the scientist belonged to. Pedro Graça was identified as the most influential scientist in the debate as was in charge of PNPAS from 2012 until 2019 and still works as a consultant for PNPAS. Regarding legitimacy, this category of stakeholders obtained the highest average legitimacy in the group. Overall, the position of Portuguese scientists on FoPL systems and particularly Nutri-Score was perceived as unclear by most of the experts:

“So, the situation is curious because we came close to adopting the Nutri-Score [in Portugal, around 2019] with strong support from consumer associations. At the time, the Portuguese scientists were rather supportive of Nutri-Score but then they became more reserved about it”. Expert 6.

In the media, we identified some declarations from Portuguese scientists expressing their concerns with some elements of Nutri-Score’s algorithm without positioning themselves as opposed to it:

“Simplified nutrition labelling is a public health measure that can represent very important gains for literacy and health of the population, and we have always positioned ourselves in favour of its implementation in Portugal (including the Nutri-Score option). However, these tools need algorithm improvements and constant reflection by nutritionists, so that one of the basic principles for the implementation of any public health measure – first do no harm – can be guaranteed”. [45]

A few Portuguese scientists clearly positioned themselves in favour of Nutri-Score by signing the European petition in favour of Nutri-Score as it was highlighted by expert 3 and expert 4:

“The previous director (Pedro Moreira, 2014-2018) of FCNAUP (Faculty of Nutrition and Food Sciences in Porto) is one of the Portuguese scientists that signed the EU petition in favour of Nutri-Score.”

However, most of scientists in Portugal remained rather neutral/discrete regarding their positions on Nutri-Score.

Finally, media had one of the lowest visibilities among the stakeholders identified which could explain a relatively low attributed power on the issue.

The media coverage on the topic of FoPLs in Portugal was low according to expert 1:

“Media do not really talk about Nutri-Score, they started talking about it when we [consumer association] did our campaign last year, because we contacted them to talk about it. This year [2021] they started a bit more but it is not something that you see a lot. [...]So it’s beginning to be a topic, for instance the Professional Order of Nutritionists they went to the TV to talk about Nutri-Score (RTP and Porto Canal) and companies like Auchan, Aldi, Nestlé they also have information about Nutri-Score on their websites. I also spoke on TVI”.

On the media side, all experts agreed that the topic of Nutri-Score was not highly covered. The main media that could have some influence in debate if they would publish an article on FoPLs were the following: Pùblico, Espresso, Jornal de Noticias for the main newspapers and RTP, SIC and TVI for the main TV channels. In October 2021, the weekly magazine Visão published a detailed article on Nutri-Score, highlighting the lack of discussion on the subject in Portugal and exposing the pros and cons of the system [46].

##### Demanding stakeholders: low power, low legitimacy

The last group of stakeholders were considered as having lower power and lower legitimacy on the issue of FoPLs in Portugal by the eight experts. These actors can be qualified as demanding stakeholders. They were represented by two economical stakeholders: the retail sector and the food industry sector. Nevertheless, it is important to mention that there was a high variability of power level among individual stakeholders within a category.

This was particularly the case for the food retailers in Portugal that are represented by two main groups: the Sonae Group (25% of market share [32], Additional file 1: Table S4) and Jeronimo Martins (19% of market share [32], Additional file 1: Table S4) for which experts attributed a high visibility and a high power (higher than the agriculture sector).

Continente seems to have quite an influence in the debate on FoPL in Portugal according to the experts:

“There has been a constant opposition from Continente which is powerful in Portugal, Continente is powerful in Portugal both on an economic level, but also politically, the political contacts of Continente are very powerful”. Expert 6.

“And because on this topic, we [Portuguese retailer] were the first one to adopt a FoPL. So, they [the Government] listen to our experience on how we manage it and with the consumer. So, I think we have a little bit more power to influence than the other ones, because we implemented it lots of years ago to inform the costumer. I think we have a little bit more legitimacy to talk about this”. Expert 5.

Expert 8, part of DGS, also mentioned Continente, reinforcing its influence in the Portuguese debate:

“Because in terms of the retailer sector, you have one of the market leaders that adopted the MTL in 2009 so it’s difficult for them to say we are changing”.

Moreover, several marketing studies showed that Continente was perceived by Portuguese consumers as “a recognizable and reliable brand” and as “one of the most trusted brands in Portugal” [32].

It is interesting to note that the two main Portuguese retailers have opposed positions on FoPLs: Continente, the main supermarket chain belonging to Sonae, has been implementing MTL on its brand since 2009, whereas Jeronimo Martins decided to implement Nutri-Score for their brand Pingo Doce in 2021. Some international food retailers such as Auchan, Intermarché, Aldi and so on, established in Portugal, support Nutri-Score. Other retailers such as Minipreço have remained neutral. These mixed positions among Portuguese retailers could explain why no public declaration from APED (Portuguese Association of Retailing Companies) was identified. The nonsupport of Continente regarding Nutri-Score may be explained by their own position on Nutri-Score that is seen as a loss of information for their consumers compared with MTL (since Nutri-Score is a summary indicator contrary to MTL that is nutrient specific) [37]. Expert 6 evoked even a sentimental value since the long history of the brand with MTL. Finally, expert 5 mentioned the economic cost for a change of FoPL that has not been officially adopted by the European Commission yet provoking a sense of uncertainty. The influence of Continente on internal stakeholders could also have been manifested in the past when the Portuguese government wanted to adopt MTL at a national level until the Parliament suggested to consider a different FoPL in 2018. Nevertheless, MTL remained in the debate as it was the main FoPL compared with Nutri-Score in Portuguese scientific studies or internal reports.

Finally, regarding the food industry sector in Portugal, it is interesting to note that experts attributed it the lowest average power with some nuances according to the experts:

“The food industry has almost no power on the government, because we are dependent from the Agriculture and Economic Ministries, we are in the middle, the Agriculture Ministry is mainly concerned about farmers, and the Economy looks at food industry as a completely secondary concern, so we are in the middle. In FIPA (Federation of Portuguese Agri-Food Industries), we are trying as a company to have a higher representation. In FIPA, we have Nestlé, Unilever, Lactalis, etc. so we have these big companies in the board of directors. Maybe it’s the food industry that is not well organized enough to have a voice, because food industry is an important sector in Portugal. […] Maybe we could have the food industry in favour of Nutri-Score, but the main thing we all agree on is that we want a European FoPL. We say to the Portuguese government, just have a European system, for economic reasons. Even for local companies that export to other countries. We [companies] are in favour of Nutri-Score. It’s in our interest, they are things that could be better in the algorithm, but we think it’s the best system that we have. The food industry in Portugal is in large majority in favour Nutri-Score”. Expert 2.

Their role in the debate was also highlighted by several experts:

“All the companies involved in Nutri-Score in Portugal (Nestlé, Auchan, Danone) form a local coalition in Portugal, bring awareness on the topic, put pressure on the government”. Expert 3 and expert 4

No public position from FIPA, representing the food industry, was mentioned by the experts. Among the ones publicly in favour of Nutri-Score, mainly the multinational Nestlé and Danone, were mentioned by the experts as well as the Portuguese company PescaNova. Sumol Compal came out as the Portuguese company with the most power and as rather in favour of Nutri-Score although it has not publicly communicated on it. Expert 1 noticed a change regarding the food industry position compared with the previous experience with MTL:

“FIPA plays a big role, they haven’t expressed their opinion yet on Nutri-Score, but what I see contrary to MTL, is that they don’t oppose Nutri-Score. They might not oppose Nutri-Score”.

To summarize the position of economic stakeholders in Portugal, expert 8 gives us an internal point of view:

“FIPA (Federation of Portuguese Agri-Food Industries) and APED (Portuguese Association of Retailing Companies) are more involved in this discussion because they have more processed foods than CAP (Confederation of Farmers in Portugal). However, CAP doesn’t really support any FoPLs. The Ministry of Agriculture represents CAP. So, CAP is not so involved in the debate but they still have an influence. APED and FIPA, it’s difficult to understand their positions, because they have different associate partners that have different position on Nutri-Score inside, but I think that now all of them are supporting Nutri-Score but I am not sure”.

### Comparison with consumers perception

**Fig. 5:**
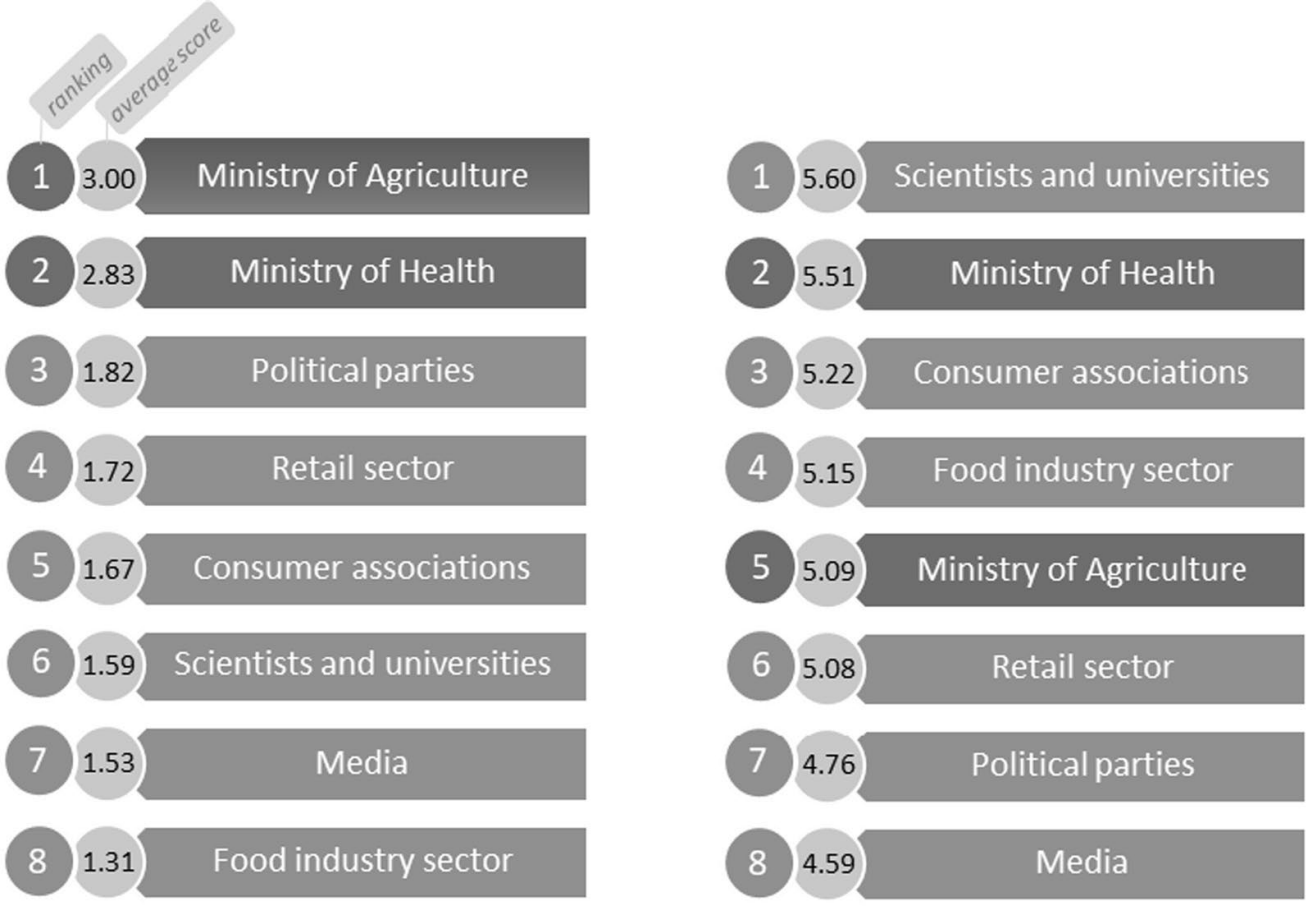
Internal (dark grey) and external (light grey) stakeholders power ranking by experts (left) and consumers (right)

**Fig. 6:**
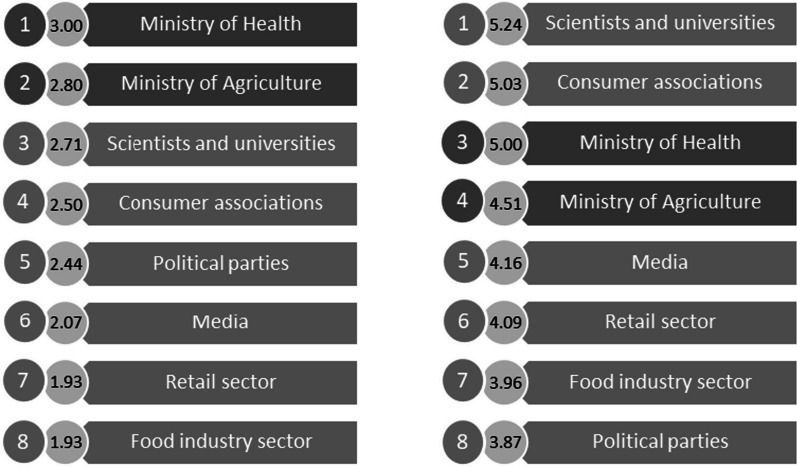
Internal (dark grey) and external (light grey) legitimacy ranking by experts (left) and consumers (right)

In a final analysis, we compared the relative power and legitimacy of stakeholders as perceived by experts with consumers’ views of these same attributes (Figs. 5, 6).

Scientists and universities were considered as both highly legitimate and powerful on the issue of FoPLs by Portuguese consumers (first position) when experts gave them also a high legitimacy (third position) but with low power (sixth position). Consumer associations were considered in the same way than scientists and universities by consumers and experts. In terms of internal stakeholders, they were placed at the top of the experts ranking whereas in the consumers, they occupied the middle of the ranking. For the experts, the Ministry of Agriculture was seen as the most powerful stakeholder on the issue of FoPLs implementation in Portugal, but in terms of legitimacy, the Ministry of Health was above. Regarding consumers, the Ministry of Health was perceived as the most powerful (second position) and legitimate (third position) internal stakeholder on the issue by consumers. Political parties were seen as having the capacity to act to implement a FoPL (third position) by experts when consumers thought the opposite (seventh position). In terms of the perceived power of the economical stakeholders, experts considered that the retail sector (fourth position) had more relative power compared with the food industry (seventh position) when for consumers, it was the opposite (fourth position for the food industry versus sixth position for the retail sector). In terms of legitimacy of the economical stakeholders, experts and consumers views were consistent with a rather low perceived legitimacy on the issue. For the media, experts and consumers ranking were also coherent. They were seen as having a low power in the debate of FoPL and a rather low legitimacy (just above the economic stakeholders).

## Discussion

### Main findings

The study of the context of the political decision in Portugal in relation with front-of-pack label implementation was a first step to analyze the situation of nonproblem in regard to the nonadoption of a national FoPL in Portugal. The proposal phase of the adoption of the MTL system at the national level carried by the Portuguese Ministry of Health only ended in 2018 when parliament suggested the analysis of a different FoPL. This turning point triggered a new set of scientific studies and technical reports testing the performance of various FoPLs in Portuguese consumers without reaching a consensus on a superior FoPL to implement. The food market environment in Portugal became divided between Continente, the main national retail operator, precursor of MTL and still using it, and other national and international food companies and retailers, as Pingo Doce, Auchan, Nestlé, Danone or Pescanova that started adopting Nutri-Score since its arrival in France in 2017. Regarding public stakeholders, the main consumer association Deco Proteste, engaged a number of actions in the promotion of Nutri-Score in Portugal, notably by organizing conferences or petitions. However, the media coverage of the issue in Portugal remained low compared with other countries such as France or Italy [12].

In terms of influence, internal stakeholders were the ones with highest capacity to act to implement a FoPL in Portugal with the Ministry of Health (DGS) and the Ministry of Agriculture (DGAV) being the central actors identified by the experts. Their opposed positions on the implementation of an interpretive FoPL in Portugal was a key finding to explain the stalling of the debate.

However, while the DGS was found to be quite supportive of the Nutri-Score format, it suggested that its algorithm could be improved [45]. The main limits identified were linked to the coherence between Nutri-Score underlying nutrient profile and the nutrient thresholds used or defined in some of the interventions part of the strategy for the promotion of healthy eating (EIPAS, 2017) such as Portugal’s regulation of food advertising for children [47].

Regarding the Portuguese Ministry of Agriculture, it is interesting to note that apart from the General Directorate of Food and Veterinary (DGAV) that was cited as a key stakeholder, the General Directorate for Agriculture and Rural Development (DGADR), responsible for the promotion of the Mediterranean diet in Portugal, was identified by experts as having a very low influence or as not involved in the debate on FoPL, when in Italy its equivalent ministry, the Ministry of Agricultural Food and Forestry Policies (MiPAAF), had a high influence on the topic and the impact of Nutri-Score on the Mediterranean diet was at the centre of the Italian debate [12]. No public declaration from the DGAV was identified on the issue of FoPLs, their opposition to Nutri-Score and interpretive FoPLs in general could be identified thanks to the experts’ interviews.

In terms of external stakeholders, the Portuguese agriculture sector was classified as having some power but low legitimacy in the debate on FoPLs. We could qualify this actor as dormant or dangerous according to his capacity to act in urgency (not assessed in this study). Yet, from the experts’ analysis, its visibility was low as it was rather muted on the issue compared with Spain or Italy. Only a few public declarations were identified such as the one by the Consortium of Olive Oil which expressed its desire for a revision of Nutri-Score algorithm or an exemption for olive oil. Nevertheless, according to experts, the opposition of the agriculture sector, represented by the Ministry of Agriculture, to the Nutri-Score format remained the major barrier to the adoption of Nutri-Score in Portugal.

A majority of actors was classified as discretionary/dependent stakeholders with some legitimacy on the issue but with low power to act or to influence the decision. In this group, consumer associations and scientists were the ones with the highest legitimacy and visibility perceived by experts. In terms of position on FoPLs, media, political parties and scientists were in majority neutral while the Portuguese consumer association Deco Proteste was found to be in favour of Nutri-Score.

Finally, the stakeholders with lower relative power and legitimacy were two economical stakeholders: the retail and the food industry sector, classified as demanding stakeholders. Regarding thw Mitchell classification, we can assume that these actors possess the urgency to act in the specific case of FoPLs since they can voluntary decide to implement a format at brand level. High variability in the power level and positions on Nutri-Score were identified in the Portuguese retail sector, with the two most powerful distributors being either in favour of the MTL system (Continente) or the Nutri-Score system (Jeronimo Martins). The food industry sector seemed rather favourable for the adoption of Nutri-Score with some national food companies already engaged and no food company publicly opposed to Nutri-Score.

### Policy implications

Henry (2021) suggests three elements lead to solving a nonproblem situation: collective action, scientific expertise, and legal and judiciary rules. In terms of collective action, our analysis highlighted the mobilization of the consumer association Deco Proteste but, as mentioned in the article from Visao Saude [46], it did not lead to any public stance from the government: “What is certain is that the discussion about this [Nutri-Score] and other models of simplified labelling systems seems to be going unnoticed, even after an open letter was sent to Parliament by the consumer association [Deco Proteste]”. As an extension of mobilizations and collective action, E. Henry insists on the role of resonance chambers such as judicial or media arenas to bring issues to wider spaces and interest larger audiences. He adds that taking charge of an issue within the media arena leads by definition to a widening of the potentially interested public since journalists are able to broadcast to a large audience. As shown in the results, the main newspapers or TV channels in Portugal did not highly cover the issue of the implementation of FoPLs. However, the fact that the implementation of FoPLs is a measure linked to prevention may reduce its urgency in the debate and may therefore explain why citizens are less mobilized on this subject than on food safety scandals, for example, which have a direct impact on consumer health in the short term. The discrepancy between experts’ and consumers’ perceptions of the power of stakeholders indicates a biased view by consumers of the power games that can operate on the ground when a FoPL is implemented. Indeed, consumers perceived the most legitimate stakeholders on the issue as having the highest capacity to act or to influence the implementation of a FoPL (scientists and universities, Ministry of Health and consumer associations). The role of the Ministry of Agriculture seems less known in this field among consumers.

The second element is related to the creation of new forms of scientific expertise that are more open and representative of the viewpoints of different groups in society. Although there was a high mobilization of the Ministry of Health on the issue, producing a high number of technical and scientific reports to guide the political decision, we can suggest that a higher collaboration with public stakeholders such as consumer associations or associations in the field would accelerate the agenda setting of a such a measure.

The third modification of the legal and judiciary rules in the context of FoPLs would imply long-term structural changes where, for instance, the Ministry of Agriculture which is a recurrent opposed stakeholder in the implementation of FoPLs, would have less decisional and regulatory power compared with the Ministry of Health. Indeed, the lack of will to change among a few actors involved in the decision-making process is enough to maintain a situation in which their interests are preserved [13]. Portuguese nutrition experts mentioned that the particular role of the Ministry of Agriculture in the area of food labelling posed significant challenges for interventions in this area. They stated that it was then necessary to identify interventions that have the capacity to combine the interests of several ministries and to identify the economic and social gains that can be achieved with investments in prevention and promotion of healthy eating [27].

### Limitations and conclusions

The strengths and limits of this study included the selection of experts in almost all stakeholders’ categories in both public and private sectors although a higher number of respondents would have allowed for a finer evaluation of stakeholders’ relative positions. Also, the document review and interviews did not allow for the identification of the positions of the various political parties during the time period. Additional review of parliamentary discussions might have refined the analysis of the political debate around FoPLs. The use of a discrete scale to qualify the degree of power and legitimacy of stakeholders with experts allowed a finer evaluation than with a dichotomous assessment. Adding the notion of visibility of a stakeholder to its influence also refined the approach. Finally, though stakeholder theory allows to investigate the potential influence of various stakeholders, it does not provide information as to the activities conducted by the various stakeholders to influence decision. The analysis of consumer perceptions was based on a large number of participants (*n* = 1014). However, as we had to develop the questionnaire in parallel with the expert interviews, we did not include all stakeholder categories in the consumer survey, as some of them emerged during the final stages of the expert interviews. Nevertheless, the most visible stakeholders were included in the consumer questionnaire. Regarding the population sample, we aimed at approaching quotas of the Portuguese population (Eurostat, 2020 [30]); however, participants may have stronger interests in food-related topic or may live in more urbanized area as internet access is needed. We adapted the stakeholder theory to our context by removing the urgency attribute which also clarified the visual appreciation of stakeholders’ influence.

In conclusion, our analysis of the Portuguese political decision-making context regarding the implementation of a front-of-pack label, as well as the characterization of the actors involved in this public health issue, provided elements of understanding to explain the reasons leading to a situation of nonproblem. The main explanations appear to come from internal stakeholders within the Portuguese government who have had the greatest influence on the issue: the past engagement of the Ministry of Health with the interpretive, nutrient-specific MTL system, the lack of support from the Ministry of Agriculture [43] for any interpretive front-of-pack labels and the reservations expressed by the Ministry of Health about the Nutri-Score algorithm, particularly in relation to its consistency with some elements of current Portuguese dietary guidelines. In parallel, all the external stakeholders in Portugal seem to push for the adoption of a single harmonized FoPL at a national level, with a majority of them supporting Nutri-Score option except for Continente, the major retail group in Portugal, which did not publicly express its opposition to Nutri-Score but uses the MTL format since more than 10 years. However, even if the debate is still covered [48, 49], the low media coverage of the issue in Portugal as well as the lack of a common public position of the main representative of the economic sectors such as the food industry and the retail sector, may have weakened the pressure exerted by external stakeholders on the government to take a political decision on the implementation of a harmonized front-of-pack label in Portugal. Finally, the context of an imminent decision by the European Commission on the adoption of a harmonized and mandatory front-of-pack label at the European level could explain the wait-and-see position of the Portuguese government on this issue. Future studies could investigate the equilibrium between EU policy regulations and national perspectives, including further aspects such as the international strategies of large food corporations and retailers, their implication in national-specific contexts and, from governmental perspective, the alliances across countries at the EU level. Moreover, from a theoretical point of view, the addition of a historical perspective, the refinement of the definition of stakeholders and the comparison of influence as perceived by experts and the population could improve the explanatory power of the SHT for understanding public decisions. In future research, this framework could be applied to other public policies to increase the applicability of stakeholder theory, for example, to improve understanding of lobbying and corporate influence of the tobacco or the alcohol industry on public health decisions.

### Supplementary Information

**Additional file 1: Figure S1.** Classification of the main Front-of-Pack nutrition Labels in Europe. **Table S1.** Main articles retrieved from the document review. Legend: scientific papers (green); grey literature (grey); press articles (pink). References can be found in the main paper’s references. **Table S2.** Interview guide. **Table S3.** Variables used to test the perceived power and legitimacy of stakeholders involved in the implementation of a Front-of-Pack nutrition Label among Portuguese consumers. **Table S4.** Main food retailers in Portugal in 2013 (Picoto and Henriques, 2018).

## Data Availability

The datasets used and/or analyzed during the current study are available from the corresponding author on reasonable request.
