# Supplementary material for: Understanding the influence of stakeholders on the implementation of front-of-pack nutrition labelling in the absence of public debate: the case of Portugal
Source: Health Res Policy Syst. 2024 Feb 7;22:20. doi: 10.1186/s12961-023-01065-8 (PMC10848542; doi:10.1186/s12961-023-01065-8)
Supplement: Supplementary file 1 — Additional file 1: Figure S1. Classification of the main Front-of-Pack nutrition Labels in Europe. Table S1. Main articles retrieved from the document review. Legend: scientific papers (green); grey literature (grey); press articles (pink). References can be found in the main paper’s references. Table S2. Interview guide. Table S3. Variables used to test the perceived power and legitimacy of stakeholders involved in the implementation of a Front-of-Pack nutrition Label among Portuguese consumers. Table S4. Main food retailers in Portugal in 2013 (Picoto and Henriques, 2018). [file 12961_2023_1065_MOESM1_ESM.docx]

**Figure S1:** Classification of the main Front-of-Pack nutrition Labels in Europe

**
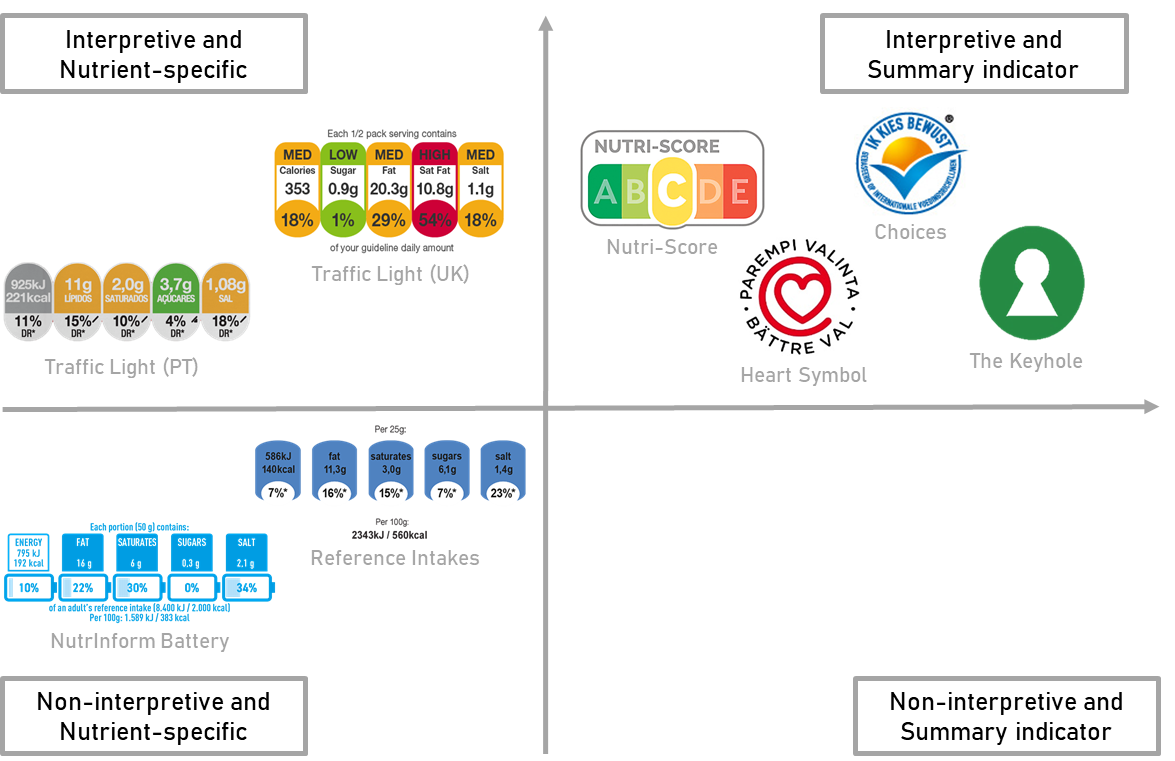
**

**Table S1:** Main articles retrieved from the document review

| **Authors** | **Title** | **Date** | **Journal** |
| --- | --- | --- | --- |
| Goiana-da-Silva, F.; Cruz-e-Silva, D.; Nobre-da-Costa, C.; Nunes, A.M.; Fialon, M.; Egnell, M.; Galan, P.; Julia, C.; Talati, Z.; Pettigrew, S.; et al. | Nutri-Score: The Most Efficient Front-of-Pack Nutrition Label to Inform Portuguese Consumers on the Nutritional Quality of Foods and Help Them Identify Healthier Options in Purchasing Situations. | 2021 | Nutrients |
| Santos, O.; Alarcão, V.; Feteira-Santos, R.; Fernandes, J.; Virgolino, A.; Sena, C.; Vieira, C.P.; Gregório, M.J.; Nogueira, P.; Graça, P. | Impact of Different Front-of-Pack Nutrition Labels on Online Food Choices. | 2020 | Appetite |
| Goiana-da-Silva, F.; Cruz, D.; Gregório, M.J.; Nunes, A.M.; Calhau, C.; Hercberg, S.; Rito, A.; Bento, A.; Cruz, D.; Almeida, F. | Nutri-Score: A Public Health Tool to Improve Eating Habits in Portugal. | 2019 | Acta medica |
| Graça, P.; Gregório, M.J.; Freitas, M. da G. | A Decade of Food and Nutrition Policy in Portugal (2010–2020). | 2020 | Portuguese Journal of Public Health |
| Graça, P.; Gregório, M.J.; de Sousa, S.M.; Brás, S.; Penedo, T.; Carvalho, T.; Bandarra, N.M.; Lima, R.M.; Simão, A.P.; Goiana-da-Silva, F.; et al. | A New Interministerial Strategy for the Promotion of Healthy Eating in Portugal: Implementation and Initial Results. | 2018 | Health Research Policy and Systems |
| Daniela Costa Teixeira | Not everything green is so good. Nutri-Score's shortcomings. *(translated from Portuguese)* | 2021 | VISÃO SAÚDE |
| Pedro Graça, Maria João Gregório | Simplified nutritional labeling and the need for reflection. *(translated from Portuguese)* | 2020 | Pensar Nutrição |
| Carlos Caldeira | Casa do Azeite. Mariana Matos: Nutri-score algorithm "clearly penalises 100% natural products".  *(translated from Portuguese)* | 2021 | Agricultura e mar |
| Store Magazine | DECO conference discusses labelling systems.  *(translated from Portuguese)* | 2020 | Store Magazine |

Legend: scientific papers (green); grey literature (grey); press articles (pink). References can be found in the main paper’s references.

**Table S2:** Interview guide

| **Theme** | **Question(s)** |
| --- | --- |
| Representation of the public health problem | - Do you think Portugal is a country where people are healthy? - Is obesity, overweight a problem in Portugal? In children? - And compared to other European countries? |
| Opinion on public health measures in Portugal | - Regarding nutrition, are there any public health measures carried out by Portugal that you are aware of? - What do you think about the management of nutritional/obesity issues in Portugal? - Specificities compared to other European countries? |
| Portuguese relationship to food | - What relationship do the Portuguese have with food? - Is the moment of eating still seen as something "sacred"? (e.g.: taking time to eat at noon, with other people, cooking, etc.) - Are there any traditional foods that are well known in Portugal (and sold in supermarkets)? - Food distribution model: many supermarkets (size, distribution)? Small shops, markets? |
| *Show the Portuguese stakeholder grid by explaining the notions of power, legitimacy, urgency*  *Have the interlocutor react on the listed stakeholders + rating of the power, legitimacy, urgency of these PPs (scale from 1 to 3) + possible differences with the French system, are there any missing?* | |
| The actors of public health/food in Portugal | - Who are the main actors involved in food-related public health in Portugal? - The main public institutions? - The main consumer associations? Their role? - The main industrial groups, their roles? - The media? Differences compared to France? - Their power, legitimacy, urgency in the application of a public health measure such as the adoption of a nutritional logo? |
| Nutri-Score | - What do you think about nutritional logos? Do you think that nutritional logos can be useful in the fight against overweight/obesity in Portugal? - What do you think about the Nutri-Score? - Do you remember when you first heard about the Nutri-Score? - Do you know who created the Nutri-Score? - Who is talking about the Nutri-Score in Portugal (the media, politicians (one party in particular?), the government, etc.)? - Is the population aware of the Nutri-Score? |
| Other questions ideas | - Are there other public health strategies that you think are more effective than the Nutri-Score in the fight against overweight and obesity and in improving nutrition education? - Insist on differences with France? |

**Table S3:** Variables used to test the perceived power and legitimacy of stakeholders involved in the implementation of a Front-of-Pack nutrition Label among Portuguese consumers

|  | ***Strongly Disagree***  **1** | **2** | **3** | ***Neither agree nor disagree***  **4** | **5** | **6** | ***Strongly Agree***  **7** |
| --- | --- | --- | --- | --- | --- | --- | --- |
| ***Perceived legitimacy*** | | | | | | | |
| [Stakeholders] have legitimacy to influence the decision to implement a FoPL on food products |  |  |  |  |  |  |  |
| [Stakeholders] are legitimately involved in the debate on the implementation of a FoPL on food products |  |  |  |  |  |  |  |
| [Stakeholders] seek to defend their own interests |  |  |  |  |  |  |  |
| [Stakeholders] seek to defend the interests of consumers. |  |  |  |  |  |  |  |
| ***Power to act*** | | | | | | | |
| [Stakeholders] have the capacity to act to implement a FoPL on food products |  |  |  |  |  |  |  |
| [Stakeholders] have the power to influence the decision to implement a FoPL on food products |  |  |  |  |  |  |  |

**Table S4:** Main food retailers in Portugal in 2013 (Picoto and Henriques, 2018)

| Food retailer | Supermaket names | Online operations | Food retail market share | Number of stores | Selling area (m^2^) | Earnings before interest and tax (millions of €) |
| --- | --- | --- | --- | --- | --- | --- |
| **SONAE MC** | Continente | Yes | 25% | 376 | 457,000 | 82,8 |
| **Jerónimo Martins** | Pingo Doce | No | 19% | 270 | 584,000 | 239,0 |
| **Intermarché** | Intermarché | No | 9,5% | 232 | 320,000 | 35,4 |
| **Lidl** | Lidl | No | 9,1% | 238 | 233,000 | 46,6 |
| **Mini Preço** | Dia  Mini Preço | No | 7% | 591 | 221,000 | 49,3 |
| **Auchan** | Jumbo  Pão de Açucar | Yes | 6,3% | 32 | 197,000 | 15,9 |
